# RETRACTION: Cholesterol Promotes Colorectal Cancer Growth by Activating the PI3K/AKT Pathway

**DOI:** 10.1155/jo/9854580

**Published:** 2025-09-09

**Authors:** Journal of Oncology

RETRACTION: C. Wu, M. Wang, and H. Shi, “Cholesterol Promotes Colorectal Cancer Growth by Activating the PI3K/AKT Pathway,” *Journal of Oncology* 2022 (2022): 1515416, https://doi.org/10.1155/2022/1515416.

The above article, published online on 29 April 2022 in Wiley Online Library (https://wileyonlinelibrary.com), has been retracted by John Wiley & Sons Ltd.

The retraction has been agreed upon following an investigation into concerns raised by a third party, which identified overlap with another article published by a different author group.

More specifically, concerns have been identified regarding the similarity of the Cho cell plate as shown in Figure 4(b) to the cell colony as shown in Figure 4(b) (left) of [1]. Similar features have also been identified between the top Control cell plate in Figure 4(b) to the siRNF6 colony depicted in Figure 2(b) of [1].

As a result, the conclusions of the current article are considered unreliable.

The authors have been informed of the retraction but did not provide a response.
